# The Differential Effects of Anger and Sadness on Intertemporal Choice: An ERP Study

**DOI:** 10.3389/fnins.2021.638989

**Published:** 2021-07-08

**Authors:** Tao Suo, Xuji Jia, Xiyan Song, Lei Liu

**Affiliations:** ^1^Institute of Psychology and Behavior, Institute of Cognition, Brain, and Health, School of Education, Henan University, Kaifeng, China; ^2^Key Research Base of Humanities and Social Sciences of Ministry of Education, Academy of Psychology and Behavior, Tianjin Normal University, Tianjin, China; ^3^Department of Psychology, College of Teacher Education, Ningbo University, Ningbo, China; ^4^Center of Group Behavior and Social Psychological Service, Ningbo University, Ningbo, China

**Keywords:** anger, sadness, emotion, intertemporal choice, ERP

## Abstract

Previous research has taken a valence-based approach to examine the carryover effects of incidental emotions on intertemporal choices. However, recent studies have begun to explore the effects of specific emotions on intertemporal choices. In this study, we investigated how anger and sadness influenced intertemporal choices using event-related potentials (ERPs). Behavioral results showed that, compared with neutral prime, anger prime was associated with more preference for delayed rewards, whereas sad prime did not change individuals’ choice preference. Specifically, anger prime yielded a shorter response time than sad prime for the difficult-to-select choices. ERP results found that, compared with neutral and sad primes, anger prime elicited larger P1 in the fronto-central and parietal areas, larger P2 in the fronto-central area, and larger P3 in the parietal area during the evaluation stage. These findings suggest that there are differential carryover effects of anger and sadness on intertemporal choice. This study provides enlightenment on the significance of understanding how incidental emotions affect individuals’ intertemporal choices.

## Introduction

Intertemporal choices require people to trade-off between costs and benefits that occur at different points in time (e.g., would you prefer $20 today or $40 in 30 days?) (Frederick et al., 2002). Decisions about investments, spending, savings, mortgages, relationships, education, and diet all involve intertemporal trade-offs. These decisions not only affect an individual’s health, wealth, and overall happiness, but also decide the economic prosperity of nations (Frederick et al., 2002). Although intertemporal choices are important and ever-present, people often make choices in a certain emotional state. Research on the influence of emotions on decision-making is based on two perspectives (Lerner et al., 2015). One is the influence of emotions induced by the characteristics of decision-making events (i.e., integral emotion) on decision-making behavior, and the other is the influence of emotions that are not directly related to decision-making events (i.e., incidental emotion) on decision-making behavior. Increasing studies have shown that incidental emotions, which are carried over from one situation to another, influence intertemporal choices that are unrelated to that incidental emotion (Loewenstein, 2000; Raeva et al., 2010; Lerner et al., 2015; Lempert and Phelps, 2016).

Most research has taken a valence-based approach to examine the carryover effects of incidental emotions on intertemporal choices. Some studies found that people in a negative emotional state made more immediate choices, while people in a positive emotional state made more far-sighted ones. For example, Wang and Liu (2009) found that participants in a negative emotional state would be willing to accept the smaller and immediate rewards, whereas those in a positive emotional state would be willing to wait and accept the larger and delayed rewards. This was consistent with the findings of Guan et al. (2015). Their results showed that, compared with neutral and positive emotions, negative emotion induced individuals to choose the smaller and immediate rewards. Other studies suggested that there were opposite effects of negative and positive emotions on intertemporal choices. That is, the negative state encouraged people to combat impatience, whereas the positive state made them more present biased. For example, Li and Xie (2012) found that when the decision conflict was high, participants in negative emotional states would select the larger and delayed rewards more often, relative to participants in positive and neutral emotional states. Moreover, Hirsh et al. (2010) found that extraverted individuals were more likely to prefer a smaller and immediate reward over a larger and delayed reward when first put in a positive state. In summary, these studies suggest that the effects of emotional valence on intertemporal choices are inconsistent, and specific emotions of the same valence may have different effects on intertemporal choices.

Anger and sadness are two kinds of negative emotions that are common in life. Previous studies showed that anger and sadness were associated with different facial expressions (Ekman, 2007), central nervous system activity (Phelps et al., 2014), brain hemispheric activation (Harmon-Jones and Sigelman, 2001), autonomic responses (Levenson et al., 1990), and cognitive appraisals (Smith and Ellsworth, 1985; Lerner and Keltner, 2000). The appraisal-tendency framework suggests that incidental emotions are related to specific appraisals. These appraisals reflect the core meaning of the event that elicits each emotion and determine the influence of specific emotions on judgment and decisions (Smith and Ellsworth, 1985; Lerner and Keltner, 2001). Research showed that anger was related to high certainty and control and sadness was related to medium certainty and low control (Smith and Ellsworth, 1985). Simultaneously, certainty and control were related to the cognitive factors of intertemporal choices (e.g., the unknown risk and low control of delayed options). For example, regarding intertemporal choices, studies found that longer waiting time for rewards meant greater risk of not getting it, with delayed rewards considered risky and unsafe (Benzion et al., 1989; She et al., 2010). Studies have also shown that control played an important role in intertemporal choices; that is, compared to high control, individuals with low control were more inclined to choose immediate rewards (Berns et al., 2007; Hare et al., 2009; Figner et al., 2010; Casey et al., 2011). Therefore, anger and sadness may have differential influences on intertemporal choices.

To date, few studies have examined the effects of anger and sadness on intertemporal choices. Lerner et al. (2013) investigated the effects of sadness and disgust, induced by emotional clips on intertemporal choices. Their results showed that, relative to the neutral state participants, the sad state participants preferred smaller and immediate rewards for payment. However, the disgust state participants were not more impatient than the neutral state participants. Recently, Zhao et al. (2017) explored the effects of state and trait anger on intertemporal choices. The results showed an interactive effect between state and trait anger on choice preference. When individuals were in a temporary state of high anger, high-trait anger individuals tended to prefer small and immediate rewards, compared with low-trait anger individuals; however, in a temporary state of low anger, low-trait anger individuals tended to prefer small and immediate rewards. Furthermore, their results found that the individuals’ preference for small and immediate rewards was associated with less risk taking for decisions made under uncertainty, indicating that the larger and delayed rewards in intertemporal choice were risky. The above research used different emotion-inducing materials and then separately examined the influence of specific negative emotions on intertemporal choices. This study aimed to examine the effects of anger and sadness, which were primed by emotional faces, on intertemporal choices in an experiment.

Event-related potentials (ERPs) have high temporal resolution and can provide the temporal dynamics of the neural activity of intertemporal choice in milliseconds. ERP research on intertemporal choices mainly found three components. The first component, P2, is the primary evaluation component; it reflects the advanced perceptual processing of certain attributes (Kranczioch et al., 2003; Boudreau et al., 2008). Regarding an intertemporal choice task, compared with a small delayed reward amount and a short delay time, a large delayed reward amount and a long delay time induced larger P2 (Gui et al., 2016; Wu et al., 2016). These findings indicate that individuals can process the reward amount and time attributes in the early stages of decision-making. The second component, P3, is considered a measure of motivation intensity in decision-making, reflecting the influence of decision-making information on motivation level (Nieuwenhuis et al., 2005; Wu and Zhou, 2009). ERP research on intertemporal choices found that when the immediate options were presented, high-anxiety individuals had a greater P3 than low-anxiety individuals; additionally, when delayed options were presented, low-anxiety individuals had a greater P3 (Xia et al., 2017). The third component is LPP. The amplitude of LPP reflects the level of motivational participation in stimulus processing and the amount of attentional resource allocation (Delplanque et al., 2004). Regarding the intertemporal choice task, a long delay time induced a smaller LPP than a short delay time (Gui et al., 2016). Speculating from the above content, P2 reflects the processing of the advanced attributes of decision-making options, while P3 and LPP reflect the evaluation of the degree of motivation for decision-making options. Therefore, this study examined whether there were differences in the three ERP components affecting the influence of anger and sadness on intertemporal choices.

To our knowledge, most studies investigated the impact of incidental emotion on intertemporal choice by adopting a between-subjects design, in which participants were induced to a specific enduring mood state by reading autobiographical stories, watching film clips, or conducting different cognitive tasks (Wang and Liu, 2009; Hirsh et al., 2010; Raeva et al., 2010; Li and Xie, 2012; Lerner et al., 2013). However, a few studies adapted a within-subjects design to study the impact effect, in which participants were induced to a transient emotional state by emotional cues (i.e., emotional pictures or faces), during the completion of the intertemporal choice task (Luo et al., 2012; Guan et al., 2015). This study investigated the effects of anger and sadness on intertemporal choice by using ERPs. Based on the appraisal-tendency framework, anger and sadness may have different effects on intertemporal choices based on their sense of certainty and control. This study assumes that, compared with neutral and sad emotions, the high certainty and control of anger makes individuals choose large delayed rewards. This study recorded and then analyzed the ERP components in the evaluation stage, during the intertemporal choice task, thereby examining the process mechanisms of anger and sadness that influence intertemporal choices.

## Materials and Methods

### Participants

Twenty healthy volunteers participated in the study (mean age = 19.30 ± 1.17 years, 12 females). All participants were right-handed and had normal or corrected-to-normal vision. Participants provided written informed consent and were paid for participation. This study was approved by the local ethics committee at the Department of Psychology, Ningbo University.

### Stimuli Selection

Facial images were selected from the Taiwanese Facial Expression Image Database (TFEID; Chen and Yen, 2007). The TFEID consisted of posed facial expressions (neutral, anger, contempt, disgust, fear, happiness, sadness, and surprise) by actors in training; the actors received written instructions of each emotional expression according to Ekman’s intervention. Angry, sad, and neutral facial images were selected with direct gaze, front view, and high intensity. There were 30 pictures each of angry, sad, and neutral facial images; the ratio of male to female in each type of facial image was 12:18.

### Intertemporal Choice Task

We administered a modified version of the intertemporal choice task (McClure et al., 2004; Kable and Glimcher, 2007), in which participants made a series of hypothetical choices between small and immediate rewards and larger and delayed rewards. The small immediate amount was one of the three reward amounts (¥18, ¥19, and ¥20). The larger delayed option was constructed using one of the three delays (7, 15, and 30 days) and 1 of the 10 add-percentages of the immediate reward (7 days: 10, 15, 20, 30, 50, 70, 90, 120, 150, and 180%; 15 days: 15, 20, 30, 50, 70, 90, 120, 150, 180, and 215%; 30 days: 20, 30, 50, 70, 90, 120, 150, 180, 215, and 250%). The immediate reward amounts and time delay of the delayed rewards were of orthogonal design.

**FIGURE 1:**
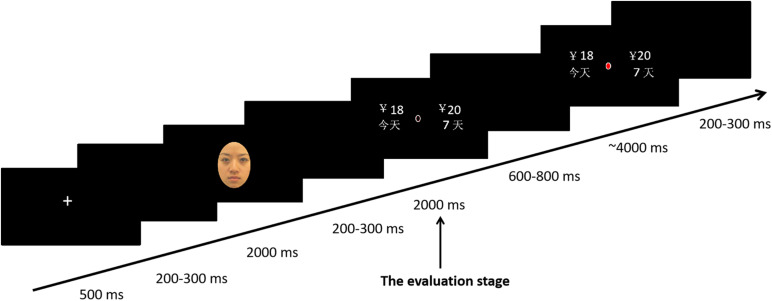
Sequence of events in a single trial of the modified intertemporal choice task.

In each trial, a white fixation was shown for 500 ms, signaling the start of the trial. After 200–300 ms of random blank, facial images were presented for 2,000 ms. Then, the immediate and delayed offers were shown for 2,000 ms, followed by 600–800 ms of random blank space. During the choice stage, the red color of the central cue instructed subjects to make a choice within 4,000 ms. The locations of the immediate and delayed options were randomly assigned (left or right) on each trial and were counterbalanced across trials. Participants were instructed to press the “F” key to denote a left-side choice or the “J” key to denote a right-side choice (see Figure 1).

### EEG Recording and Analysis

Electroencephalograms (EEGs; NeuroScan Inc.) were recorded from 64 electrodes, which were mounted on an elastic cap. The horizontal electrooculogram (HEOG) and vertical electrooculogram (VEOG) were recorded as well. The left mastoid was the online reference electrode. All electrode impedances were maintained below 5 kΩ. All signals were sampled at 500 Hz and band-pass filtered within a 0.05–100 Hz frequency range. During off-line analyses, all EEG signals were re-referenced to the mean of the left and right mastoids. The EEG data were low-pass filtered below 30 Hz (24 dB/oct). Ocular artifacts were removed from the data using a regression procedure (Semlitsch et al., 1986). Trials containing EEG sweeps with amplitudes exceeding ± 70 mV were excluded.

For the evaluation stage ERPs, the EEG was averaged by channel and time window, from 200 ms before to 1,000 ms after the evaluation options presentation. According to grand-mean ERP waveforms and relevant literature (Li et al., 2012; Gui et al., 2016; Wu et al., 2016; Xia et al., 2017), we measured the peak amplitude of P1 (70–120 ms) and the mean amplitudes of P2 (200–250 ms), P3 (320–400 ms), and LPP (550–900 ms) components over the fronto-central (Fz, FCz, and Cz) and parietal areas (Pz and CPz).

### Statistics

First, based on the study of McClure et al. (2004), this study distinguished the choices into easy-to-select choices and difficult-to-select choices. The easy-to-select choices included 7 days (10–20%, 150–180%), 15 days (15–30, 180–215%), and 30 days (20–50%, 215–250%). The difficult-to-select choices included 7 days (30–120%), 15 days (50–150%), and 30 days (70–180%). The behavioral measures (the rate of immediate choices and response time) were analyzed using a two-way ANOVA, with emotion type (anger vs. sad vs. neutral) and task difficulty (easy vs. difficult) as the within-subject factors.

For the ERP components time-locked to the evaluation stage, a two-way ANOVA was used, with emotion type (anger vs. sad vs. neutral) and task difficulty (easy vs. difficult) as the within-subject factors. A Greenhouse–Geisser correction was applied to all ANOVAs when necessary. The significance levels were set at *p* < 0.05, and the marginal significance levels were set at 0.05 ≤ *p* < 0.1.

## Results

In this section, we reported the behavioral and ERP results of the valuation stage. Two subjects were excluded due to severe artifacts in the EEG data, resulting in 18 participants being included for the ERP analysis. For the sake of brevity, the statistic effects that were not significant were omitted.

### Behavioral Results

**FIGURE 2:**
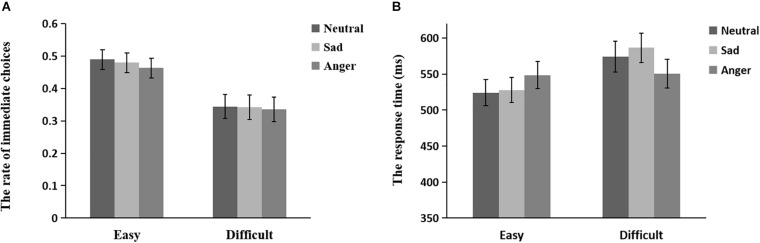
The means and SEs of the rate of immediate choices **(A)** and the response time **(B)** in anger, sad, and neutral prime conditions for the easy-to-select choices and the difficult-to-select choices.

Figure 2 shows the means and SEs of the rate of immediate choices and the response time in anger, sad, and neutral prime conditions for easy-to-select and difficult-to-select choices.

For the rate of immediate choices, the main effect of emotion type was significant, *F*(2, 38) = 3.42, *p* = 0.048, η^2^ = 0.153. The *post hoc* test showed that the anger prime yielded a lower rate of immediate options than neutral (*p* = 0.023) and sad primes (*p* = 0.071), with no significant difference between sad and neutral primes (*p* = 0.880). The main effect of task difficulty was significant, *F*(1, 19) = 9.90, *p* = 0.005, η^2^ = 0.343, suggesting that participants making difficult-to-select choices had a higher rate of delayed options than those making easy-to-select choices (also see Figure 2A).

For response time, the main effect of task difficulty was significant, *F*(1, 19) = 8.64, *p* = 0.008, η^2^ = 0.313, suggesting that it was significantly longer in the difficult-to-select choices than in the easy-to-select choices. The interactive effect of emotion type and task difficulty was significant, *F*(2, 38) = 4.51, *p* = 0.020, η^2^ = 0.192. For the difficult-to-select choices, the anger prime yielded a significantly shorter response time than the sad prime, whereas there were no significant differences between neutral and anger primes and between neutral and sad primes (*p*s > 0.100). For the easy-to-select choices, the anger prime yielded a longer response time than the neutral prime (*p* = 0.089), and there were no significant differences between sad and anger primes and between sad and neutral primes (*p*s > 0.100). Furthermore, the response time in the difficult-to-select choices was significantly longer than that in the easy-to-select choices for sad (*p* = 0.001) and neutral primes (*p* = 0.024), whereas there was no significant difference between the easy-to-select and difficult-to-select choices for the anger prime (*p* = 0.592) (also see Figure 2B).

### ERPs Results

**FIGURE 3:**
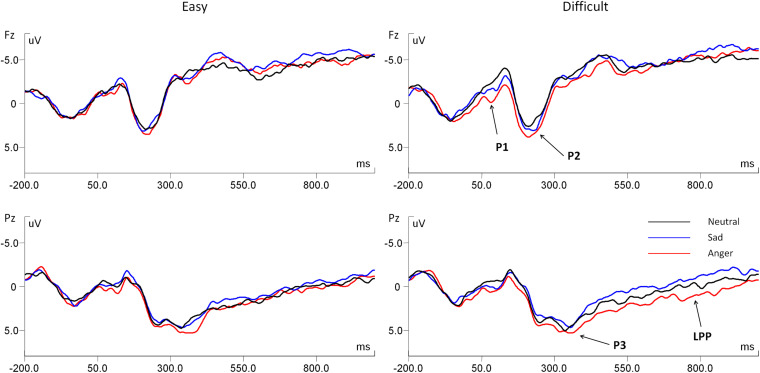
Grand average ERPs during the evaluation stage at Fz and Pz in the anger, sad, and neutral prime conditions for the easy-to-select choices and the difficult-to-select choices.

**FIGURE 4:**
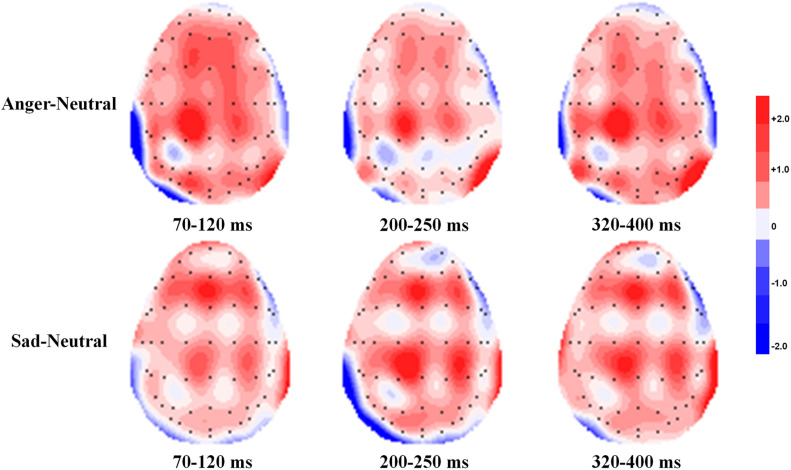
Topographic maps depicting voltage differences for the anger prime condition minus the neutral prime condition and the sad prime condition minus the neutral prime condition in the time range of P1 (70–120 ms), P2 (200–250 ms), and P3 (320–400 ms) during the evaluation stage.

Figure 3 shows the grand average ERPs during the evaluation stage at Fz and Pz in the anger, sad, and neutral prime conditions for the easy-to-select and difficult-to-select choices. Figure 4 shows the topographic maps depicting voltage differences for the anger minus the neutral prime conditions, and the sad minus the neutral prime conditions in the time range of P1 (70–120 ms), P2 (200–250 ms), and P3 (320–400 ms), during the evaluation stage.

*P1(70–120 ms)*

For the fronto-central P1, the main effect of emotion type was significant, *F*(2, 34) = 7.17, *p* = 0.005, η^2^ = 0.297. The *post hoc* test showed that the anger prime evoked a larger P1 than neutral (*p* = 0.007) and sad primes (*p* = 0.012), whereas there was no significant difference between sad and neutral primes (*p* = 0.138). For the parietal P1, the main effect of emotion type was marginally significant, *F*(2, 34) = 3.00, *p* = 0.079, η^2^ = 0.150. The *post hoc* test showed that the anger prime evoked a larger P1 than neutral (*p* = 0.036) and sad primes (*p* = 0.080), whereas there was no significant difference between sad and neutral primes (*p* = 0.331).

*P2(200–250 ms)*

For the fronto-central P2, the main effect of emotion type was marginally significant, *F*(2, 34) = 3.04, *p* = 0.070, η^2^ = 0.152. The *post hoc* test showed that the anger prime evoked a larger P2 than neutral (*p* = 0.011) and sad primes (*p* = 0.086), whereas there was no significant difference between sad and neutral primes (*p* = 0.611). For the parietal P2, there were no significant main and interactive effects of emotion type and task difficulty (*p*s > 0.100).

*P3(320–400 ms)*

For the fronto-central P3, there were no significant main and interactive effects of emotion type and task difficulty (*p*s > 0.100). For the parietal P3, the main effect of emotion type was marginally significant, *F*(2, 34) = 2.77, *p* = 0.086, η^2^ = 0.140. The *post hoc* test showed that the anger prime evoked a larger P3 than neutral (*p* = 0.019) and sad primes (*p* = 0.067), whereas there was no significant difference between sad and neutral primes (*p* = 0.841).

*LPP(550–900 ms)*

For the fronto-central and parietal LPP, there were no significant main and interactive effects of emotion type and task difficulty (*p*s > 0.100).

## Discussion

By combining different emotional (anger, sadness, and neutral) primes with the intertemporal choice task, this study found that anger and sad primes were differentiated in both their effects on intertemporal choice and the temporal dynamics of neural activity during intertemporal decision-making. Behavioral results showed that the anger prime (relative to neutral prime) was associated with more preference for delayed rewards. Specifically, the anger prime yielded a shorter response time than the sad prime for the difficult-to-select choices. ERP results found that the anger prime (relative to neutral and sad primes) elicited larger P1 in the fronto-central and parietal areas, P2 in the fronto-central area, and P3 in the parietal area during the evaluation stage.

This study found that, compared with the neutral prime, the anger prime encouraged individuals to prefer more delayed rewards. Based on the appraisal-tendency framework, an emotion can have strong influences on intertemporal choices that relate to the appraisal theme of the emotion. In this study, certainty and control are central dimensions that distinguish anger from other negative emotions (Weiner et al., 1982; Averill, 1983; Smith and Ellsworth, 1985). For example, anger is related to a sense of certainty in individuals that they have enough information to feel confident in their judgment and a high coping potential that they have the capacity to deal with the situation (Smith and Ellsworth, 1985; Tiedens and Linton, 2001; Berkowitz and Harmon-Jones, 2004). Moreover, previous studies found that certainty and control were conceptually related to intertemporal choice. For example, intertemporal choices were associated with unknown risk (e.g., perceiving delayed rewards as risky and uncertain) and impulsivity (the temptation of immediate rewards) (Benzion et al., 1989; Berns et al., 2007; Hare et al., 2009; Figner et al., 2010; She et al., 2010; Casey et al., 2011). Therefore, the sense of certainty and high coping potential induced by anger can make people combat the temptation of immediate rewards, in preference for delayed rewards. Consistent with this view, this study suggested that angry individuals intended to choose larger and delayed rewards.

Generally, the response time of the intertemporal choice task can be considered as an index of the struggle between immediate and delayed options (Wu et al., 2016). This is consistent with the finding that the response time in the difficult-to-select choices was longer than that in the easy-to-select choices, indicating that there were more conflicts in the difficult-to-select choices. In this study, there was an interesting result, that for difficult-to-select choices, the anger prime yielded a shorter response time, compared with the sad prime. One possible explanation is that, compared with sadness, anger increased individuals’ sense of certainty and control (Smith and Ellsworth, 1985; Tiedens and Linton, 2001), and then experiencing a sense of certainty and control motivated them to quickly make a decision from the difficult-to-select choices.

In this study, we also observed emotional prime effects on the temporal dynamics of neural activity, similar to behavioral results. First, the anger prime (relative to neutral and sad primes) elicited a larger P1 during the evaluation stage. Previous research found that P1 was sensitive to physical stimulus factors and indexed early sensory processing within the extra-striate visual cortex (Gonzalez et al., 1994; Clark and Hillyard, 1996; Luck et al., 2000). Furthermore, although P1 has been considered to be purely stimulus-driven and exogenous, there are recent findings that P1 can be influenced by high-level information, such as emotional valence, threat-related information, semantic knowledge, and reward processing (Eimer and Holmes, 2002; Smith et al., 2003; Keil et al., 2005; Rahman and Sommer, 2008; Schacht et al., 2012). For example, P1 was found to be larger for unpleasant than pleasant pictures, indicating that unpleasant pictures engaged more attentional processing than pleasant pictures (Smith et al., 2003). This study further suggested that P1 can be influenced by emotion type during the evaluation stage. That is, the anger prime makes individuals pay more automatic and fast attention to processing the intertemporal option information, compared with neutral and sad primes.

Second, the anger prime (relative to neutral and sad primes) elicited a larger P2 in the fronto-central area during the evaluation stage. Previous ERP studies on decision-making showed that the frontal P2 might reflect stimulus evaluation and quick assessment (Potts et al., 2006; Boudreau et al., 2008; Nikolaev et al., 2008; Chen et al., 2009). Specifically, ERP studies on intertemporal choice found that a larger frontal P2 was associated with a longer time delay and a larger reward amount during intertemporal decision-making, indicating the initial valuation of time and reward information (Gui et al., 2016; Wu et al., 2016). Consistent with those studies, the larger P2 in the anger prime condition might be related to the quick evaluating process involved in the information of reward amount and time delay during intertemporal decision-making.

Third, the anger prime (relative to neutral and sad primes) elicited a larger P3 in the parietal area during the evaluation stage. A previous ERP study on intertemporal choice showed that the P3 elicited by the immediate option was larger in the high trait anxiety group than in the low trait anxiety group. In addition, the P3 elicited by the delayed option was enhanced in the delayed decision condition for low trait anxiety, compared to high trait anxiety participants, indicating that the P3 is reflected to index the motivational significance of different options (Xia et al., 2017). This was consistent with the study of Li et al. (2012) that showed that an enhanced P3 has been found in individuals who show a larger delay discounting effect, indicating stronger motivations to pursue immediate over delayed rewards. In addition, the P3 was also regarded as an index to examine various advanced cognitive processes (i.e., memory encoding and updating, evaluation and stimulus categorization, and making decisions under complex social context) (Kok, 2001; Polich, 2007; Chen et al., 2009; Paynter et al., 2009; Mathes et al., 2012). In this study, our results found that the P3 amplitudes in the anger prime condition were significantly larger than in neutral and sad prime conditions, suggesting that more attentional and controlled cognitive processing resources are required in the anger prime condition and that participants had stronger motivations to select the delayed options.

This study found that, compared with neutral and sad emotions, anger, which is related to high certainty and control, made individuals choose large, delayed rewards. The study further found that anger in individuals with high certainty and control motivated them to place more attention and motivation to evaluating the choices, displaying larger P1, P2, and P3 amplitudes. If a sense of certainty and control enhances the tendency to delay gratification in intertemporal choices, positive emotions that are related to certainty and control senses should have the same effect. Future research should independently manipulate the certainty and control dimensions as well as the valence of emotions. Furthermore, it should explore whether specific emotions affect intertemporal choices through the certainty and control dimensions, while excluding their valence. In addition to using the appraisal-tendency framework to explain how specific emotions affect intertemporal choices, some researchers also used the construal level theory and the perceived-time-based model to explain this process. Specifically, the construal level theory suggested that any object or event in the environment can be characterized at different construction levels (Liberman et al., 2002): High and low construction levels. Under high-level construction, people tended to characterize long-term events, while under low-level construction, people specifically characterize recent events. The construal level theory highlights that specific emotions affect the individuals’ construction level and then affect the individuals’ choice preference (Wang and Liu, 2009). In addition, Zauberman et al. (2009) proposed the perceived-time-based model to explain the cognitive mechanism of intertemporal choices. They found that the discounting rate in intertemporal choices decreased as the objective delayed time increased; the reason may be that individual perception of future time is biased (Zauberman et al., 2009). The perceived-time-based model suggests that specific emotions affect the individuals’ subjective perception of future time and then affect the individuals’ choice preference. It remains unclear whether anger and sadness affected the individuals’ construction level or subjective perception of the future time and then affected choice preference in intertemporal choices, which need further research.

This study has some limitations. First, anger and sadness were induced by emotional faces in this experiment. Although this method is one of the most common and effective methods to induce specific emotions, future research can use different emotion induction methods, including watching a video clip that induces anger and sadness, or experiencing an angry or sad event live, to determine the generality of the results of this study. Second, the sample size in this study may be too small; follow-up research needs to further expand the sample size. Moreover, the samples of this study are all composed of college students. In the future, a diverse sample (e.g., individuals of different ages) will be needed to evaluate the external validity of this study and further expand the conclusions of this experiment. Third, this study examined the influence of anger and sadness on intertemporal choices in the gain situation. Intertemporal choices involve two types of situations: Gains and losses. A large number of studies in the field of intertemporal choices have shown that the internal cognition and neural mechanisms of loss- and gain-based intertemporal choices are not equivalent, and the results obtained in the gain situation cannot be generalized to the loss situation (Gehring and Willoughby, 2002; Xu et al., 2009; Mitchell and Wilson, 2010). Therefore, it was necessary to study the influence of anger and sadness on intertemporal choices for both gain and loss situations. Fourth, previous studies found that different levels of emotional arousal also have different effects on intertemporal choices (Fedorikhin and Patrick, 2010; Sohn et al., 2015). For example, Sohn et al. (2015) examined the impact of high arousal of positive and negative emotions on intertemporal choice. The results showed that, compared with neutral emotional states, individuals tend to choose smaller timely rewards in high positive and negative emotional states. Future research needs to investigate the impact of interactions between specific emotions and arousal on intertemporal choices.

## Conclusion

In conclusion, this study found that anger and sadness had differential effects on intertemporal choices. That is, the anger prime motivated individuals to prefer delayed rewards, whereas the sad prime did not change the preference for intertemporal choice. The ERP results were different in P1, P2, and P3, during the evaluation stage. These findings suggest that, relative to neutral and sad primes, the anger prime motivates individuals to place more attention and motivation to evaluate their choices and makes them choose the delayed rewards.

## Data Availability Statement

The original contributions presented in the study are included in the article/supplementary material, further inquiries can be directed to the corresponding author/s.

## Ethics Statement

The studies involving human participants were reviewed and approved by a local ethic committee at the Department of Psychology, Ningbo University. The patients/participants provided their written informed consent to participate in this study.

## Author Contributions

LL and TS designed the study and wrote the manuscript. XJ collected and analyzed the data and XS revised the manuscript. All authors approved the version to be published.

## Conflict of Interest

The authors declare that the research was conducted in the absence of any commercial or financial relationships that could be construed as a potential conflict of interest.
